# Case report: Significant response to PD-L1 inhibitor after resistance to PD-1 inhibitor in an advanced alpha-fetoprotein-positive gastric cancer

**DOI:** 10.3389/fonc.2022.962126

**Published:** 2022-10-27

**Authors:** Liyu Wang, Ying Feng, Anquan Huang, Jianming Shi, Qinying Zhang, Fan Zhu, Bin Lv, Fen Guo, Tianming Zou, Luyao Zhang

**Affiliations:** ^1^ The Affiliated Suzhou Hospital of Nanjing Medical University, Suzhou Municipal Hospital, Gusu School, Nanjing Medical University, Suzhou, Jiangsu, China; ^2^ Department of Orthopaedics, Union Hospital, Tongji Medical College, Huazhong University of Science and Technology, Wuhan, China

**Keywords:** positive alpha-fetoprotein, gastric cancer, PD-1, immunotherapy, chemotherapy

## Abstract

Alpha-fetoprotein-positive gastric cancer (AFPGC) is a type of gastric cancer with a high degree of malignancy. The disease is more common in the elderly, with a high prevalence in males and generally atypical clinical manifestations. For advanced patients, the current treatment options are limited and, to date, few cases of advanced AFPGC have been treated successfully with conventional chemotherapy. With the development of molecular biology and immunology, tumor immunotherapy offers more therapeutic options to patients with advanced gastric cancer. This study describes a case of advanced gastric cancer in a young woman with a high blood alpha-fetoprotein (AFP) level (>54,000 ng/mL). The patient showed initial promising results when programmed cell death-1 (PD-1) inhibitor treatment was combined with chemotherapy after systemic chemotherapy failed. When the disease progressed again after 129 days, adjustment of the treatment regimen to Atezolizumab in combination with Irinotecan and Surufatinib capsules achieved partial remission (PR). There were no immune-related pneumonia, myocarditis, or other adverse effects observed. The patient currently has an overall survival of more than 14 months. This case demonstrated that switching from PD-1 inhibitor to programmed cell death-Ligand 1 (PD-L1) inhibitor therapy may overcome potential resistance. It providing a reference for immunotherapy of patients with AFP-positive advanced gastric cancer.

## Introduction

Gastric cancer with elevated serum AFP levels confirmed histopathologically after excluding hepatitis, cirrhosis, hepatocellular carcinoma, germ-cell malignancy, and other illnesses that may cause AFP is referred to as AFPGC (1). AFPGC is considered to be one of the most aggressive tumor subtypes in gastric cancer. It has been reported that AFPGC accounts for 2.3%~7.1% of total gastric cancer in Asian countries and about 15% of total gastric cancer in western countries (2). It was reported that patient age, TNM stage and curable surgery were found to be associated with overall survival. The younger AFPGC patients are prone to have a more detrimental prognosis. A high level of AFP is an independent prognostic risk factor for gastric cancer because AFP is not only a product of the tumor but also plays a crucial role in proliferation, apoptosis, and angiogenesis of AFPGC cells (3). AFP has been reported to have a suppressive effect on lymphocyte transformation, to enhance tumor cell proliferation through the HGF and c-Met pathway (4), and to increase angiogenesis *via* Vascular Endothelial Growth Factor (VEGF) expression (5).

According to the World Health Organization (WHO) (2019) classification of gastric cancer, serum AFP may be elevated in several types of gastric adenocarcinoma, such as hepatoid adenocarcinoma and gastric adenocarcinoma with enteroblastic differentiation. Previous research has found that serum AFP levels are an independent risk factor impacting patient survival (6), and there is currently no effective treatment for AFPGC.

Here, we report the case of a 37-year-old woman suffering from gastric cancer with an extremely high expression of serum AFP level (>54,000 ng/mL). The patient showed initial promising results when PD-1 monoclonal antibody (mAb) treatment was combined with chemotherapy after systemic chemotherapy failed. When the disease progressed again after 129 days, adjustment of the treatment regimen achieved PR.

## Case description

**Figure 1:**
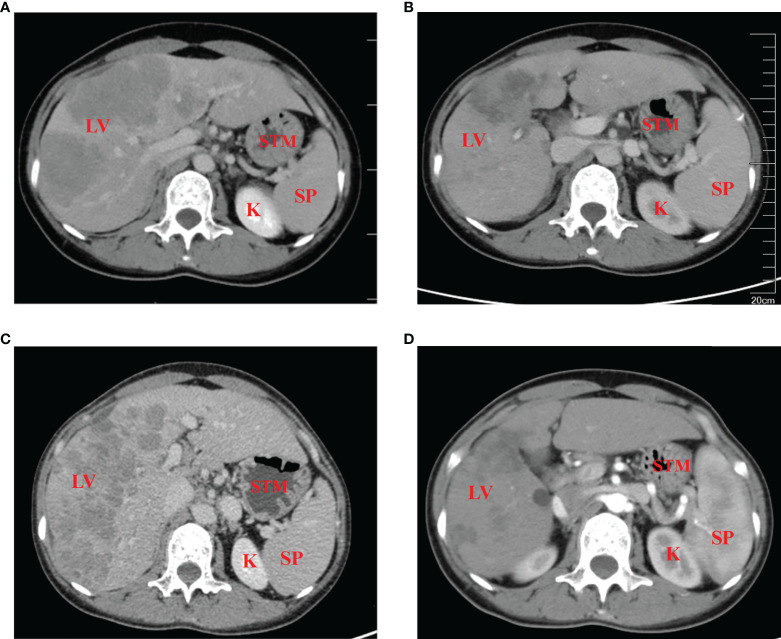
Contrast-enhanced CT scans of the patient **(A)** prior to, and **(B)** 5 months after the initiation of therapy. **(C)** At 6 months post-diagnosis, the curative effect was evaluated as progressive disease, with no effect of third-line therapy of Tislelizumab combined with Apatinib. **(D)** The disease returned to partial remission after treatment with the fourth-line treatment. LV, liver; STM, stomach; SP, spleen; K, kidney.

**Figure 2:**
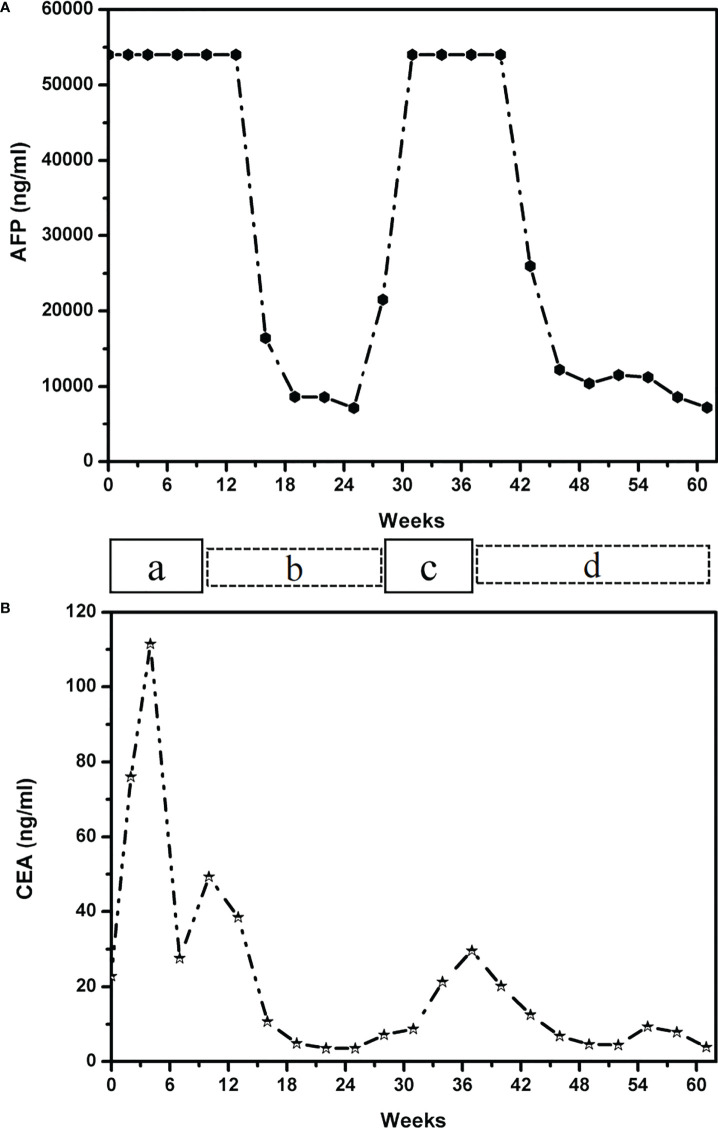
Graph depicting the patient’s therapeutic course and serum tumor marker of AFP levels **(A)** and CEA level **(B)**. The patient received the following treatments: a, paclitaxel liposome plus S-1 (tegafur/gimeracil/oteracil potassium); b, Tislelizumab combined with Oxaliplatin; c, Tislelizumab combined with Apatinib; d, Atezolizumab in combination with Irinotecan and Surufatinib capsules. AFP, alpha-fetoprotein; CEA, carcinoembryonic antigen.

**Figure 3:**
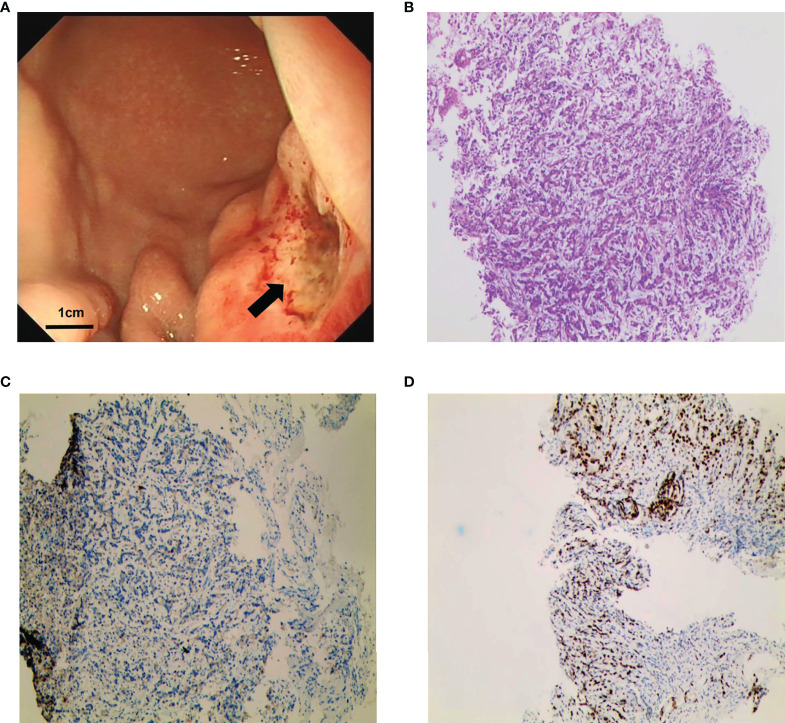
Gastroscopy and gastric biopsy pathology results. **(A)** Image of gastroscopy results, showing a tumor located at the body of the stomach (as indicated by the arrows). **(B)** Histopathological examination revealed poorly differentiated gastric adenocarcinoma (hematoxylin and eosin staining; magnification, ×100). **(C)** Negative staining for HER2 (magnification, ×100). **(D)** Positive staining for Mut L homolog 1 (MLH1) (magnification, ×100).

In January 2021, a 37-year-old woman complained of right upper abdomen discomfort and mild tenderness in a prone posture. She also had felt a palpable mass in the right upper abdomen. Physical examination revealed an enlarged liver with a bulge and palpable mass in the right abdomen. The lower margin of the enlarged liver was at the right midclavicular line about the level of the navel. The patient had tenderness in the liver area, and the Numeric Rating Scales (NRS) score was 2 points. The patient had no tumor-related family history and denied having a chronic liver illness (hepatitis, cirrhosis, and primary liver cancer) or combined reproductive system tumors. After hospitalization, an enhanced Computed Tomography (CT) examination was performed to further evaluate the patient’s condition, which showed there were multiple nodular lesions in the liver with local gastric wall thickening (**Figure 1A**). Among the tumor markers, AFP levels were significantly elevated to more than 54,000 ng/ml (normal level: < 12 ng/ml), and the serum carcinoembryonic antigen (CEA) levels were 22.82 ng/ml (normal range: < 5 ng/ml) (**Figures 2A, B** **)**. Gastroscopy revealed a giant crateriform ulcer within the stomach body (**Figure 3A**), and pathological examination showed a poorly differentiated adenocarcinoma. Immunohistochemical labeling revealed the presence of proficient mismatch repair (pMMR)/microsatellite stability (MSS) as well as deficiencies in Human Epidermal growth factor Receptor 2 (HER2) and EBV-encoded RNA (EBER) hybridization (**Figures 3B–D**). The patient was diagnosed with poorly differentiated gastric adenocarcinoma with liver metastases (HER2 negative). Meanwhile, the patient was a gastric cancer with elevated serum AFP levels. Hepatitis, liver cirrhosis and other diseases that may lead to elevated AFP were excluded. Therefore, this patient was an advanced AFPGC patient.

The patient received first-line chemotherapy in a two-drug combination regimen of paclitaxel liposome for injection and S-1 (tegafur/gimeracil/oteracil potassium). After two sessions of therapy, the disease status was stable, as determined by imaging. Then, in the third course, the S -1 dose was increased to 60 mg orally twice daily from day 1 to day 14 and continued to be combined with 210 mg paclitaxel liposome for injection. However, following the third session of chemotherapy, the patient reported that the upper abdominal distension was worse than before, and her AFP levels was consistently greater than 54,000 ng/ml. After communicating with the patient and completing the informed consent form for immunotherapy, the patient began to receive 200mg Tislelizumab injections in combination with 200mg Oxaliplatin treatment on April 17, 2021, every 21 days as a course. After two courses of treatment, the patient’s abdominal distension was relieved. An abdominal CT scan indicated that the liver lesions were smaller than before, and gastric wall thickening was reduced. The imaging evaluation showed that the disease status was stable. The original regimen was continued for two courses, and imaging was performed after the fourth course to assess PR (**Figure 1B**). Regular reexamination showed a progressive decrease in AFP level to 7169 ng/ml and the CEA level dropped to within the normal range (**Figures 2A, B** **)**. The patient’s symptoms of pain in the liver area had been relieved and subsequently the analgesic drugs were stopped.

At 6 months post-diagnosis, the serum level of AFP had increased again to 21,520 ng/ml (**Figure 2A**). At the same time, CT revealed that the liver lesions had increased in size again (**Figure 1C**). The curative effect was evaluated as progressive disease (PD). Then, the patient was given third-line therapy of Tislelizumab combined with Apatinib 250 mg once a day for 3 cycles from August 23, 2021 to October 5, 2021. Unfortunately, the patient did not respond well to this treatment, and radiographic assessments continued to show progression of the disease. Therefore, a fourth-line treatment of Atezolizumab in combination with Irinotecan and Surufatinib capsules was considered from October 27, 2021. After 2 cycles of treatment, CT evaluation showed that the efficacy achieved PR again (**Figure 1D**), blood examination showed a progressive decrease in AFP level to 12,238 ng/ml (**Figure 2A** **)**. Considering this positive response, another 5 cycles of Atezolizumab in combination with Irinotecan and Surufatinib therapy were conducted with the last treatment on March 23, 2022, and the efficacy was assessed as sustained PR.

The therapy was well tolerated by the patient during the whole treatment process, and the main adverse reactions were bone marrow suppression (leucopenia of 2 degrees) and digestive tract reactions (nausea and vomiting), which improved after treatment with Granulocyte-colony-Stimulating-Factor (G-CSF) and enhanced antiemetic therapy. There were no immune-related pneumonia, myocarditis, or other adverse effects observed. The patient currently has an overall survival of more than 14 months.

## Discussion

AFPGC is a special and rare subtype of gastric carcinoma. The histological diagnosis of AFPGC is more common in poorly differentiated adenocarcinoma, which is strongly associated with larger tumor volume, deeper serous membrane infiltration, and higher levels of invasion, lymph node, and liver metastasis (7). AFPGC has a poorer prognosis than AFP-negative gastric cancer. The median survival of advanced AFPGC is about 9.3 months (8).

There is currently no standard treatment for this type of gastric cancer. However, early radical resection and active postoperative adjuvant chemotherapy have been shown to enhance the prognosis of AFPGC patients. For patients who have lost the chance of surgical treatment in the late stage, there is little literature on treatment. At present, the chemotherapy plan is mostly referred to as common gastric cancer, but the efficacy is worse than that of common gastric cancer. The median Overall Survival (OS) of patients is 9.3 months, and the 5-year survival rate is less than 20% (9). In 2018, Wang reported the efficacy of different chemotherapy regimens in 105 patients with advanced AFPGC, and the Overall Response Rate (ORR) of the platinum-based triple regimen was 56.1%, which was better than that of double-regimen (26.3%) (10). in 2021, Li reported that three patients received oral Apatinib 500 mg once a day combined with XELOX (Oxaliplatin/Capecitabine). One patient reached PR with progression-free survival(PFS) for more than 13 months in the first line treatment, and the other for 7 months, the third one had PFS for 3 months (11). Arakawa also reported that Ramucirumab targeting Vascular Endothelial Growth Factor Receptor 2 (VEGFR2) had some efficacy in AFPGC patients with an OS of 16 months (12). In this case, the patient was confirmed to have multiple liver metastases at the time of diagnosis and lost the opportunity of surgical treatment. The patient failed to benefit from the first-line two-drug combined chemotherapy, and did not respond to Apatinib, which confirmed that the therapeutic effect was worse than that of ordinary gastric cancer, and the patient’s treatment options were limited.

Immunotherapy has demonstrated some efficacy in the treatment of advanced gastric cancer in recent years. The checkmate-649 study (13) showed that the combination of Nivolumab and chemotherapy increased the duration of PFS and OS in patients with Combined Positive Score (CPS) ≥ 5 and CPS ≥ 1 compared with chemotherapy alone, and a statistical difference was seen in the entire population (13.8 months versus 11.6 months, HR = 0.80). The results of ATTRACTION-4 showed that the median PFS time (10.5 months: 8.3 months, HR=0.68) and ORR (57.5% vs 47.8%, P=0.0088) were significantly better than those of chemotherapy alone. Furthermore, this was population-wide research with no molecular marker selected (14). The results of the Checkmate-649 and ATTRACTION-4 studies confirm the role of immunochemotherapy in the first-line treatment of gastric cancer. However, few studies have reported on the efficacy of advanced AFPGC immunotherapy. The patient described here, was a young female with MSS molecular typing and HER2 negative expression. The patient came with extensive liver metastasis and a significant tumor load. If the first therapy was ineffective, the patient might expect to live for only a short time. This patient did not benefit significantly from the three courses of first-line chemotherapy, and the AFP level was continuously above the critical value of 54,000 ng/ml. Due to the limited pathological tissue obtained by gastroscopy, the detection of PD-1 expression level could not be carried out. In the case of unknown PD-1 expression level, we chose Tiralizumab immunotherapy combined with oxaliplatin chemotherapy as the second line treatment. After 2 cycles of treatment, the AFP level decreased significantly and liver lesions shrank. PR was achieved after 4 cycles of treatment. The patient was relatively sensitive to PD-1 mAb combined with chemotherapy, but the remission period was short. Previous data suggest that Apatinib targeted anti-angiogenesis therapy was an effective way to overcome AFPGC. But unfortunately, this patient did not show efficacy in the treatment regimen of third-line. Sorafenib is one of the anti-VEGF drugs that has been reported to be effective (15, 16), but has not been reported in the treatment of AFPGC. In this case, the patient was changed to the PD-L1 ab therapy, when PD1 mAb resistance progressed. Surprisingly, it was found that the treatment effect was very good, and achieved immunotherapy re-challenge successfully. It demonstrated that switching from PD-1 inhibitor to PD-L1 inhibitor therapy may overcome potential resistance. The main mechanism is that PD-L1 mAb not only inhibit the PD1-PDL1 pathway, but also can activate DC cells and T cell functions by blocking the co-inhibition of B7.1 and PD-L1. At present, the disease is still in remission, there were no obvious adverse reactions during the whole treatment and it was well tolerated by the patient. The rise and fall in serum AFP levels during treatment were found to be positively correlated with the patient’s condition, and elevation of serum AFP level may be detected prior to appearance of symptoms and imaging detection. Therefore, measuring the serum AFP levels as a follow-up marker is an important means that can be used to evaluate condition changes of a patient.

With the emergence of a new generation of gene sequencing technology, gastric cancer can be divided into different subtypes, based on gene mutations. In 2014, TCGA (The Cancer Genome Atlas) reported the results of genomic mapping of 295 cases of primary gastric Cancer and established four genomic subtypes (17), including microsatellite instability (MSI), Epstein-Barr virus infection (EBV+), and tumors with low aneuploidy (GS) genomic stability and high aneuploidy (CIN) chromosomal instability. According to Arora (18), loss of heterozygosity (LOH) is common in gastric cancer and can lead to chromosomal instability and the loss of tumor suppressor genes. The majority of tumors with increased AFP expression were categorized as chromosomal instability subtypes, with a 72% median index of allele loss. This was 50% higher than normal gastric adenocarcinoma. Patients with AFPGC may respond to immunotherapy due to their unique genetic characteristics. In addition, other studies demonstrated that CIN is a driver of metastatic progression, which may partially contribute to the aggressive phenotype of AFPGC. In all, the identification of these potential tumor drivers raises the potential for tumor-specific immunotherapy.

Previous research reported that several molecular factors such as Vascular Endothelial Growth Factor-C (VEGF-C), Signal Transducer and Activator of transcription 3 (STAT3), and Hepatocyte Growth Factor (HGF) seem to over-expressed more frequently in AFPGC than in stage-matched non-AFPGC (5, 19). Chen (20) found that ANGPTL6 is an important driver gene of angiogenesis in AFPGC development. ANGPTL6 promotes endothelial cell migration and tube formation through activation of ERK1/2 and AKT pathways. ANGPTL6 knockdown inhibits cancer cell apoptosis and invasiveness. These findings provide not only effective biomarkers for diagnosis but also attractive therapeutic targets for AFPGC patients.

In conclusion, AFPGC, as a special type of gastric cancer, has a low incidence but a high degree of malignancy. Improving the understanding of this type of gastric cancer can avoid misdiagnosing it as common gastric adenocarcinoma and underestimating its malignancy. At present, only a few cases have been reported on the conversion of PD-L1 mAb after the progress of PD-1 mAb therapy, and there is still a lack of large-scale prospective studies. However, there are still many open questions, for example, how to choose the treatment plan after the progress of PD-L1 mAb therapy? How to optimize the immune combination therapy model? Despite the need of further studies to tackle those questions, this case report represents an important exploration and potential breakthrough in the treatment of advanced gastric cancer with immunotherapy.

## Data availability statement

The raw data supporting the conclusions of this article will be made available by the authors, without undue reservation.

## Ethics statement

The studies involving human participants were reviewed and approved by the ethics committee of Suzhou Municipal Hospital. The patients/participants provided their written informed consent to participate in this study. Written informed consent was obtained from the individual(s) for the publication of any potentially identifiable images or data included in this article.

## Author contributions

Conceptualization: FG, JS and TZ. Attending physicians for the patient: YF and LZ. Writing—original draft: LW. Editing draft: AH and BL. Supervision, FG, JS, and QZ. All authors contributed to the article and approved the submitted version.

## Funding

This work was supported by grants from Special Project of Diagnosis and Treatment Technology of Key Clinical Diseases of Suzhou City (LCZX201910) and the Medical Scientific Research Project of Jiangsu Commission of Health (Grant No.Z2020003 and Z2020058). The funders of the current study had no role in study design, data collection and analysis, decision to publish, or preparation of the manuscript.

## Conflict of interest

The authors declare that the research was conducted in the absence of any commercial or financial relationships that could be construed as a potential conflict of interest.

## Publisher’s note

All claims expressed in this article are solely those of the authors and do not necessarily represent those of their affiliated organizations, or those of the publisher, the editors and the reviewers. Any product that may be evaluated in this article, or claim that may be made by its manufacturer, is not guaranteed or endorsed by the publisher.

## References

[1] Kim HJ Oh SC . Novel systemic therapies for advanced gastric cancer. J Gastric Cancer (2018) 18(1):1–19. doi: 10.5230/jgc.2018.18.e3 29629216 PMC5881006

[2] Wang D Li C Xu Y Xing Y Qu L Guo Y . Clinicopathological characteristics and prognosis of alpha-fetoprotein positive gastric cancer in chi nese patients. Int J Clin Exp Pathol (2015) 8(6):6345–55. PMC4525844 26261510

[3] Lin HJ Hsieh YH Fang WL Huang KH Li AF . Clinical manifestations in patients with alpha-Fetoprotein-Producing gastric cancer. Curr Oncol (2014) 21(3):e394–9. doi: 10.3747/co.21.1768 PMC4059802 24940098

[4] Amemiya H Kono K Mori Y Takahashi A Ichihara F Iizuka H . High frequency of c-met expression in gastric cancers producing alpha- fetoprotein. Oncology (2000) 59(2):145–51. doi: 10.1159/000012152 10971174

[5] Kamei S Kono K Amemiya H Takahashi A Sugai H Ichihara F . Evaluation of vegf and vegf-c expression in gastric cancer cells producing alpha-fetoprotein. J Gastroenterol (2003) 38(6):540–7. doi: 10.1007/s00535-002-1099-y 12825129

[6] Sun W Liu Y Shou D Sun Q Shi J Chen L . Afp (Alpha fetoprotein): Who are you in gastrology? Cancer Lett (2015) 357(1):43–6. doi: 10.1016/j.canlet.2014.11.018 25462859

[7] Li XD Wu CP Ji M Wu J Lu B Shi HB . Characteristic analysis of Á-Fetoprotein-Producing gastric carcinoma in China. World J Surg Oncol (2013) 11:246. doi: 10.1186/1477-7819-11-246 24083471 PMC3849988

[8] Liu X Cheng Y Sheng W Lu H Xu Y Long Z . Clinicopathologic features and prognostic factors in alpha-Fetoprotein-Producing gastric cancers: Ana lysis of 104 cases. J Surg Oncol (2010) 102(3):249–55. doi: 10.1002/jso.21624 20740583

[9] Bozkaya Y Demirci NS Kurtipek A Erdem GU Ozdemir NY Zengin N . Clinicopathological and prognostic characteristics in patients with afp-secreting gastric carcinoma. Mol Clin Oncol (2017) 7(2):267–74. doi: 10.3892/mco.2017.1288 PMC5532699 28781800

[10] Wang YK Shen L Jiao X Zhang XT . Predictive and prognostic value of serum afp level and its dynamic changes in advanced gastric cancer patients with elevated serum AFP. World J Gastroenterol (2018) 24(2):266–73. doi: 10.3748/wjg.v24.i2.266 PMC5768945 29375212

[11] Li N Bai C Zhang R Ma L Ren X Zhang J . Efficacy and safety of apatinib for the treatment of AFP-producing gastric cancer. Transl Oncol (2021) 14(2):101004. doi: 10.1016/j.tranon.2020.101004 33383486 PMC7777135

[12] Arakawa Y Tamura M Aiba K Morikawa K Aizawa D Ikegami M . Significant response to ramucirumab monotherapy in chemotherapy-resistant recurrent alpha-fetoprotein -producing gastric cancer: A case report. Oncol Lett (2017) 14(3):3039–42. doi: 10.3892/ol.2017.6514 PMC5588133 28928842

[13] Janjigian YY Shitara K Moehler M Garrido M Salman P Shen L . First-line nivolumab plus chemotherapy versus chemotherapy alone for advanced gastric, gastro-oesophageal junction, and oesophageal adenocarcinoma (Checkmate 649): A randomised, open-label, phase 3 trial. Lancet (2021) 398(10294):27–40. doi: 10.1016/s0140-6736(21)00797-2 34102137 PMC8436782

[14] Boku N Ryu MH Kato K Chung HC Minashi K Lee KW . Safety and efficacy of nivolumab in combination with s-1/Capecitabine plus oxaliplatin in patients with previously untreated, unresectable, advanced, or recurrent Gastric/Gastroesophageal junction cancer: Interim results of a randomized, phase II trial (Attraction-4). Ann Oncol (2019) 30(2):250–8. doi: 10.1093/annonc/mdy540 PMC6386029 30566590

[15] Koneri K Hirono Y Fujimoto D Sawai K Morikawa M Murakami M . Five-year survival of alpha-Fetoprotein-Producing gastric cancer with synchronous liver metastasis: A case report. J Gastric Cancer (2013) 13(1):58–64. doi: 10.5230/jgc.2013.13.1.58 23610720 PMC3627808

[16] Fang YU Wang L Yang N Gong X Zhang YU Qin S . Successful multimodal therapy for an ¦Á-fetoprotein-Producing gastric cancer patient with simultaneous liver metastases. Oncol Lett (2015) 10(5):3021–5. doi: 10.3892/ol.2015.3731 PMC4665269 26722283

[17] Cancer Genome Atlas Research N . Comprehensive molecular characterization of gastric adenocarcinoma. Nature (2014) 513(7517):202–9. doi: 10.1038/nature13480 PMC4170219 25079317

[18] Arora K Bal M Shih A Moy A Zukerberg L Brown I . Fetal-type gastrointestinal adenocarcinoma: A morphologically distinct entity with unfavourable prognosis. J Clin Pathol (2018) 71(3):221–7. doi: 10.1136/jclinpath-2017-204535 28814568

[19] Jia Y Liu D Xiao D Ma X Han S Zheng Y . Expression of AFP and STAT3 is involved in arsenic trioxide-induced apoptosis and inhibition of proliferation in AFP-producing gastric cancer cells. PLoS One (2013) 8(1):e54774. doi: 10.1371/journal.pone.0054774 23382965 PMC3559880

[20] Chen E Tang C Peng K Cheng X Wei Y Liu T . Angptl6-mediated angiogenesis promotes alpha fetoprotein-producing gastric cancer progression. Pathol Res Pract (2019) 215(8):152454. doi: 10.1016/j.prp.2019.152454 31146977

